# Malignant catatonia responsive to low doses of lorazepam: case report: Catatonia maligna responsiva a baixas doses de lorazepam: relato de caso

**DOI:** 10.1590/1516-3180.2014.00052608

**Published:** 2015-10-09

**Authors:** Diego Fernando Moreira Matias, Sabrina de Mello Ando, Rachel Riera, Aécio Flávio Teixeira de Góis

**Affiliations:** I MD. Resident Physician, Department of Psychiatry, Universidade Federal de São Paulo (Unifesp), São Paulo, Brazil.; II Medical Student, Universidade Federal de São Paulo (Unifesp), São Paulo, Brazil.; III MD, MSc, PhD. Rheumatologist and Professor, Universidade Federal de São Paulo (Unifesp), São Paulo, Brazil; Coordinator at Brazilian Cochrane Center, São Paulo, Brazil.; IV MD, MSc, PhD. Head, Emergency Department, Universidade Federal de São Paulo (Unifesp), São Paulo, Brazil.

**Keywords:** Catatonia, Benzodiazepines, Lorazepam, Depressive disorder, Case reports [publication type]

## Abstract

**CONTEXT:**

Catatonia can be divided into non-malignant or malignant. The latter is characterized by autonomic instability, exhibiting high fever, tachycardia and hypertension, and is regarded as a fulminant and rapidly progressive subtype.

**CASE REPORT:**

This article reports a case of malignant catatonia in a 43-year-old patient who had been presenting psychiatric disorders for the last three years. The patient was stable, maintaining mutism, immobility and autonomic abnormalities. Oral lorazepam (1 mg every eight hours) was introduced and, in a few hours, the patient became afebrile. Two days later, the patient was already responding to verbal commands.

**CONCLUSIONS:**

Early intervention with lorazepam reduced the evolution of this patient to a fatal complication. Therefore, this case report sought to show that early diagnosis and intervention reduced the occurrence of serious and irreversible clinical outcomes.

## RESUMO

**CONTEXTO:**

A catatonia pode ser dividida em não maligna ou maligna. A maligna se caracteriza pela instabilidade autonômica, exibindo febre elevada, taquicardia e hipertensão, além de ser considerada um subtipo fulminante e rapidamente progressivo.

**RELATO DE CASO:**

Este artigo relata um caso de catatonia maligna em paciente de 43 anos, com transtornos psiquiátricos há três anos. A paciente estava estável, mantendo o mutismo, a imobilidade e as anormalidades autonômicas. Foi introduzido lorazepam, via oral, 1 mg de oito em oito horas, e em algumas horas, a paciente ficou afebril. Em dois dias, já estava respondendo a comandos verbais.

**CONCLUSÕES:**

Intervenção precoce com lorazepam preveniu a evolução desta paciente para um desfecho fatal. Portanto, este relato de caso mostrou que o diagnóstico e a intervenção precoces reduziram a ocorrência de desfechos graves e irreversíveis.

## INTRODUCTION

Catatonia is a serious syndrome that is present in approximately 9.8% of hospitalized adult psychiatric patients. This syndrome should be considered as a differential diagnosis among patients with signs of mutism, immobility, rigidity and autonomic abnormalities, such as fever, increased pressure blood pressure, tachycardia, tachypnea, leukocytosis and increased kinase creatine.^1^^,^^2^^,^^3^

Studies have shown that use of benzodiazepines is effective in 70% of cases, while electroconvulsive therapy is effective in 85%. There are few case reports on malignant catatonia treated only with lorazepam, although this drug could be the initial treatment of choice, because it is simple and presents considerable response in these patients.^4^

The malignant form of catatonia is considered to be a fatal and rapidly progressive subtype. It is a rare condition that presents subtle signs and symptoms and different etiologies, and is therefore underdiagnosed. This case report seeks to encourage healthcare professionals to suspect this differential diagnosis, which would enable introduction of fast and effective treatment to prevent progression of this condition.^5^

## CASE REPORT

A 43-year-old married female patient was brought to our institution by the Emergency Medical Service, from an Integrated Psychiatric Care (Assistência Psiquiátrica Integrada) unit where she had been hospitalized for 13 days with a diagnosis of bipolar disorder, with a current depressive episode with psychotic symptoms and emotionally unstable personality disorder. She was under treatment with lithium carbonate (300 mg, three times a day), valproic acid (250 mg, three times a day), risperidone (2 mg, twice a day), diazepam (10 mg, once a day), omeprazole (20 mg, once a day) and chlorpromazine (according to medical criteria).

She was conscious the night before admission, but was found unconscious and presenting breathing difficulty the next morning. Chest compressions were performed for three minutes, which returned SatO_2_ (oxygen saturation) from 35% to 90%. No medications were found with the patient.

In the emergency room at Hospital São Paulo, the patient presented Glasgow 3, and she was tachycardic (100 bpm), with hypotension (80 x 50 mmHg), blood glucose of 125 mg/dl and SatO_2_ of 86%, but without pupil description. Flumazenil was administered, without providing any improvement, and then orotracheal intubation (OTI) was performed.

The electrocardiogram input (ECG) indicated sinus bradycardia and a long QT interval. The computed tomography (CT) scan showed no signs of bleeding or acute ischemia.

At the evaluation, the patient had hypertensive peaks (140 x 70 mmHg), hyperthermia (38.5 °C), increased creatine phosphokinase (CPK) (600 U/l to 1000 U/l), but with the absence of muscle stiffness, twitching or hyperreflexia. Nevertheless, treatment for neuroleptic malignant syndrome was administered, comprising use of bromocriptine (2.5 mg every eight hours) in a nasogastric tube for a few days, without improvement.

The urine sample was sent to the Toxicological Assistance Center, which confirmed the presence of benzodiazepines, with only qualitative results, and suggested the hypothesis of exogenous benzodiazepine intoxication, despite the chronic use of this medication for the patient.

Two days after admission, the patient was transferred to the intensive care unit (ICU), and she presented worsening of her clinical parameters. A new cranial CT scan showed diffuse edema with intracranial hypertension, and therefore treatment without using mannitol was started. After a week, another cranial CT showed no signs of abnormality. Throughout this period, the patient was being monitored by the hospital’s neurology service. After the intracranial hypertension had been resolved, a hypothesis of hypoxic encephalopathy was put forward.

The electroencephalogram (EEG) examination showed marked diffuse depression of brain electrical activity. Later, another EEG exam was performed and this showed somatosensory evoked potentials, which made it possible to infer that the patient presented cortical activity and that the EEG would be compatible with a conscious individual, but with a slight downgrade that would be compatible with use of benzodiazepine. The cerebrospinal fluid examination did not show any significant changes.

After 20 days of hospitalization, the patient had two episodes of crying, for approximately 20 minutes, expressing grief, which could not be related to pain, time or presence or absence of family.

A psychiatric assessment was requested in order to evaluate the possibility of interference of psychiatric disorders in the patient’s clinical improvement.

According to the family, for the last three years, the patient has been under psychiatric treatment for major depression, bipolar disorder and borderline personality disorder. She had previously attempted suicide with rat poison and had three times tried to slit her wrists over the last three years. She also had had five prior psychiatric hospitalizations, ranging from three to 30 days and averaging 10 days. There were also reports of command auditory hallucinations for suicide. From the history collected from the family, it was only possible to make the hypothesis of severe depressive episodes with psychotic symptoms. There were few criteria for bipolar disorder and cluster B personality disorder. The patient possibly presented a depressive syndrome, but this seemed to be less important than the current clinical situation.

There was still uncertainty regarding suicide attempts, since the patient did not have any medication, there was no reversion with flumazenil, the urine test was only qualitative, she was a chronic user of benzodiazepine drugs (BDZ) and there were medications on her prescription that could cause a drop in the level of consciousness.

Therefore, the hypotheses were organic mental disorders (hypoactive delirium) and depressive syndrome, and monitoring was maintained. The patient was stable, maintaining mutism, immobility and autonomic abnormalities. The possibility of malignant catatonia was therefore suggested and, considering the risks and benefits, oral lorazepam (1 mg every eight hours) was introduced. The psychiatric diagnosis was based on exclusion, and in this case there was a possibility of clinical improvement with medication.

After the introduction of lorazepam, the patient became afebrile after a few hours and, two days later, was already responding to verbal commands (like shaking hands and moving the upper limbs). Therefore, malignant catatonia remained a hypothesis, possibly associated with hypoxic-ischemic encephalopathy.

## DISCUSSION

Kahlbaum described catatonia in 1874 as a single clinical factor composed of motor, emotional and behavioral vegetation.^3^ In the top ten prospective studies around the world, catatonic syndrome was identified, on average, in 9.8% of admissions of adult psychiatric patients.^6^ The prevalence in psychiatric populations ranges from 6% to 38%, with an average incidence of 15% among the patients hospitalized per year.^7^

There are three forms of catatonia: retarded, excited and malignant.^3^ Differentiating the three forms was an important aim of this paper.

Catatonia can also be divided in non-malignant or malignant forms. These differ with regard to the autonomic instability presented by malignant forms, which consists of high fever, tachycardia and hypertension. Speech and thoughts are disorganized, accompanied by intense excitement, cataplexy, mutism, rigidity, stereotypy and posture, and this condition can often be fatal.^3^^,^^8^ The etiology may be neurological, metabolic or exogenous and may include schizophrenia, mood disorders, general medical conditions, substance suspension and autism, apart from idiopathic causes.^3^^,^^9^

Due to the varied clinical picture, the diagnosis of malignant catatonia may be questioned since there are other differential diagnoses that should be discarded, such as neuroleptic malignant syndrome, serotonin syndrome, malignant hyperthermia, akinetic mutism, non-epileptic seizures, incarceration syndrome, Stiff’s syndrome, Parkinson’s disease, dementia and delirium.^1^

Studies have shown that most catatonic patients present mood disorders, particularly mania. Taylor and Abrams noted that 28% of their patients with bipolar disorder exhibited catatonic features.^1^^,^^10^ Gelenger correlated catatonia with neurological and general medical conditions, believing that catatonia should be considered to be a syndrome, not a disease.^11^

Benzodiazepines and electroconvulsive therapy (ECT) are considered safe and effective for relieving catatonia.^3^ Benzodiazepines can usually relieve catatonic symptoms. Lorazepam is the drug that has been most studied and used in treating catatonia, and it can be administered orally, intramuscularly or intravenously. However, in Brazil, injectable benzodiazepines are not available.^12^

We conducted a systematic search of the literature to look for reports of cases similar to those described. The results are shown in Table 1. Some of the cases found are described below. Using the MeSH terms “catatonia” and “lorazepam” to filter the case reports, we found 90 cases in Medline. By excluding the MeSH term “Schizophrenia and Disorders with Psychotic Features” in the same search, we found 69 cases. In another search, we used the term “malignant catatonia”, which is not a MeSH term, but by using this to filter the case reports, we found 51 cases in Medline.

**Table 1.**
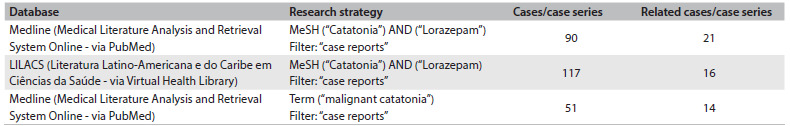
Systematic search of the literature

Search date: July 22, 2014.

Lin and Huang followed up 21 schizophrenic patients who required treatment for catatonia and received a protocol consisting of lorazepam and diazepam. The results showed that 13 patients responded in 2 hours, 18 had already responded after one day and all of them were free of catatonia one week afterwards.^3^

In the general ICU of the Ioannina General Hospital, there were seven patients with the criteria for a diagnosis of catatonic disorder. Lorazepam was administered orally (dose of 2.5-10 mg/day), because the intravenous formulation was unavailable at the hospital. Benzodiazepines are considered to be first-line treatment, and all the patients recovered successfully in 2-18 days.^13^

Ungvari et al. conducted a double-blind study with a control group receiving placebo, among a sample of 18 chronic catatonic schizophrenic patients. Lorazepam was administered at a dose of 6 mg/day for 12 weeks and it was insufficient to reduce the signs or symptoms of catatonia. This shows that the doses required for clinical improvement vary.^14^ Some cases of catatonic schizophrenia have been reported to require up to 12.5 mg/day of lorazepam for clinical stabilization.^12^^,^^15^

Tibrewal et al. showed that around one third of their patients with catatonia responded completely to a lorazepam dose of 3-6 mg/day for 3-7 days, and an improvement in the symptoms of catatonia was observed in two thirds of the cases analyzed.^4^

ECT is useful in cases that are resistant to pharmacological treatment and present prolonged catatonia.^4^^,^^16^ Petrides et al. claimed that the results from a combination of lorazepam and ECT are superior to monotherapy.^17^

Rey and Walter found 60 reports describing use of ECT in 396 young patients. Among these, serious complications were rare and the treatment led to improvement in 63% of the depression cases, 80% of the mania cases, 42% of the schizophrenia cases and 80% of the catatonia cases.^18^

## CONCLUSION

Catatonia is not a simple disease to diagnose. It requires not only knowledge among healthcare professionals, but also prompt and effective treatment. In this case report, we acknowledge that the lorazepam doses used were not high. These were administered orally and, unfortunately, there was no ECT available in our hospital. This may have affected the evolution of our patient, since there is evidence that the association of benzodiazepines with ECT has a synergistic effect. Nonetheless, early intervention with lorazepam prevented the evolution of this patient to a fatal complication. Therefore, this case report showed that early diagnosis and intervention reduced the occurrence of serious and irreversible clinical outcomes.
